# DEKR-SPrior: An Efficient Bottom-Up Keypoint Detection Model for Accurate Pod Phenotyping in Soybean

**DOI:** 10.34133/plantphenomics.0198

**Published:** 2024-06-27

**Authors:** Jingjing He, Lin Weng, Xiaogang Xu, Ruochen Chen, Bo Peng, Nannan Li, Zhengchao Xie, Lijian Sun, Qiang Han, Pengfei He, Fangfang Wang, Hui Yu, Javaid Akhter Bhat, Xianzhong Feng

**Affiliations:** ^1^ Zhejiang Laboratory, Hangzhou 311100, Zhejiang, China.; ^2^ School of Computer Science and Technology, Zhejiang Gongshang University, Hangzhou 310012, Zhejiang, China.; ^3^ Key Laboratory of Soybean Molecular Design Breeding, Northeast Institute of Geography and Agroecology, Chinese Academy of Sciences, Changchun 130102, Jilin, China.

## Abstract

The pod and seed counts are important yield-related traits in soybean. High-precision soybean breeders face the major challenge of accurately phenotyping the number of pods and seeds in a high-throughput manner. Recent advances in artificial intelligence, especially deep learning (DL) models, have provided new avenues for high-throughput phenotyping of crop traits with increased precision. However, the available DL models are less effective for phenotyping pods that are densely packed and overlap in in situ soybean plants; thus, accurate phenotyping of the number of pods and seeds in soybean plant is an important challenge. To address this challenge, the present study proposed a bottom-up model, DEKR-SPrior (disentangled keypoint regression with structural prior), for in situ soybean pod phenotyping, which considers soybean pods and seeds analogous to human people and joints, respectively. In particular, we designed a novel structural prior (SPrior) module that utilizes cosine similarity to improve feature discrimination, which is important for differentiating closely located seeds from highly similar seeds. To further enhance the accuracy of pod location, we cropped full-sized images into smaller and high-resolution subimages for analysis. The results on our image datasets revealed that DEKR-SPrior outperformed multiple bottom-up models, viz., Lightweight-OpenPose, OpenPose, HigherHRNet, and DEKR, reducing the mean absolute error from 25.81 (in the original DEKR) to 21.11 (in the DEKR-SPrior) in pod phenotyping. This paper demonstrated the great potential of DEKR-SPrior for plant phenotyping, and we hope that DEKR-SPrior will help future plant phenotyping.

## Introduction

Soybean is an agriculturally important legume crop that is rich in edible protein and oil [1]. The core objective of soybean researchers across the globe is to produce soybean cultivars with higher yields. There are many traits associated directly with soybean yield, known as yield-related traits, such as seed weight, seed shape, the number of seeds per pod, and the number of pods per plant [2–4]. Among the yield-related traits, the number of pods per plant and the number of seeds per pod are the major focuses of soybean breeders; therefore, these traits have been important targets in plant phenotyping [5–8]. Conventional manual phenotyping of these yield-related traits in soybean is often a low-throughput, labor-intensive, error-prone, and time-consuming process [9]. These challenges in manual phenotyping emphasize the need to develop an automated and high-throughput phenotyping method that facilitates the extraction and analysis of phenotypic traits from large quantities of images [10,11]. Recently, artificial intelligence-based methods, especially deep learning (DL), have been demonstrated to have a great tendency to process large image data for high-throughput phenotyping [12–15]. DL methods offer many advantages relative to conventional digital image processing techniques, particularly in the area of feature extraction [16,17]. Notably, DL models can provide more precise and resilient trait features from the raw images [18]. In summary, the DL techniques available for analyzing phenotypic traits related to soybean pods can be broadly classified into 2 categories: segmentation-based and detection-based methods.

In the case of segmentation-based methods, soybean pods are carefully selected and photographed against a controlled background. Advanced segmentation models such as YOLACT [19,20] and Mask R-CNN [21–23] are used to delineate the pod area within images to provide high-resolution data on pod counting and seed shape. In these methods, soybean pods must be removed from plants prior to image capture. These methods not only are time-consuming but also damage the structural integrity of plants, which is detrimental to breeders. Furthermore, in these methods, the pods do not overlap or touch each other in the images; hence, they are not directly applicable to in situ soybean plants. In such methods, the pods typically must be the primary subjects within the image to ensure the accuracy of segmentation; thus, the pod occupies a considerable portion of the image. However, soybean plants are very tall and narrow; hence, the pods occupy a small area of an image of a whole soybean plant. To address this situation, the resolution of the pod region must be enhanced in the input data to ensure accurate seed identification.

In detection-based methods, individual soybean pods are designated as specific targets for identification. The detection models YOLO [24,25] and SSD [26] are employed to accurately detect and enumerate the pods as well as detect the pod type; hence, they enhance the efficiency of soybean pod phenotyping. In comparison to segmentation-based methods, detection-based methods do not require the removal of pods prior to image capture. The images can be captured directly on the in situ soybean plants. However, YOLO-based algorithms are limited in detecting axis-aligned bounding boxes [27,28], whereas the soybean pods in the images may be oriented at various angles. This limitation makes it difficult for the model to accurately locate pods that are not aligned with the axes. Furthermore, soybean pods are densely packed (and may overlap each other) in in situ soybean plants, and nonmaximum suppression (NMS) filtering may eliminate closely positioned targets. This flaw can, in turn, lead to a high rate of false negatives, especially in dense areas, as pods that are too close to each other may be inadvertently filtered out by NMS, thereby failing to adequately address the issue of densely packed and overlapping soybean pods.

By considering the challenges faced by detection-based methods, particularly in the case of densely clustered pods and overlapped pods, we focus on exploring the potential of point-based detection methods such as P2PNet [29], which can overcome the challenges involving the complexities of densely and overlapped pods [30]. Point-based detection methods, such as those applied in human, pose estimation, identify individuals, and localize keypoints accurately, irrespective of the number of people present in the image [31,32]. Point-based detection methods are effective under some unfavorable conditions, such as images involving varying numbers of individuals, intersecting positions and diverse sizes; these advantages make these methods versatile and robust methods for human pose estimation and related tasks [33,34]. These methods involve 2 stages: In the first stage, keypoint coordinates are located for all individuals in the image [35], and in the second stage, individual targets are determined using keypoint grouping [36]. Heatmaps are a typical approach to locating keypoint coordinates in the first stage, whereby the point with the highest value in the local area is selected as the candidate keypoint [37]. Another approach is coordinate regression, which directly locates the coordinates of candidate keypoints in the image [38]. Although keypoint detection in human pose estimation is easy and accurate, the primary challenge is keypoint clustering during the second stage. To address this challenge, CMU-pose has a nonparametric representation known as part affinity fields (PAFs) [39], which show the position and direction of limbs to group the keypoints of an individual in the image [40]. Moreover, associative embedding (AE) [41] is another approach that generates keypoint heatmaps and label heatmaps for each body joint and subsequently assigns keypoints with similar labels to individuals [42]. DEKR [43] has recently emerged as an efficient method for estimating human poses based on keypoints. The original DEKR uses adaptive convolution to obtain a heatmap and locate the key points in the map, and it also uses the AE method for keypoint clustering, which provides good results in the case of human keypoint detection [38].

However, DEKRs are primarily used for various postures of the human body, and they are not well-suited for soybean pods. Unlike the human body, soybean pods are rigid structures, and the positions of seeds in soybean pods are relatively fixed, thus providing high uniformity in their spatial structure. This uniformity can be further enhanced and learned through neural network training, leading to considerable improvements in seed clustering accuracy. Compared to human body poses, soybean pods are more densely packed in space and exhibit relatively minor variations in shape, as well as a greater degree of feature similarity between seeds; this attribute in turn demands greater feature discrimination. In the field of facial recognition, cosine similarity has been effectively utilized to measure the similarity between feature vectors and provides additional intraclass compactness and interclass discrepancy to enhance feature discrimination [44,45]. By considering the merits and demerits of the above models or methods, we have integrated a structural prior (SPrior) block into the DEKR framework in the present study, resulting in a new model called the DEKR-SPrior (disentangled keypoint regression with structural prior) model. The DEKR-SPrior model incorporates cosine similarity to bolster the discriminability between pods, thus removing the limitations of representational learning by calculating the similarity between the channel features of seeds.

Moreover, a small proportion of soybean pods are present in the original full-sized images captured from soybean plants, and enhancing the resolution of the pod region within the input image is an effective method for improving performance [46,47]. To this end, we cropped the original image into several subimages and then used these cropped images as the training set of the input dataset in the model, followed by integrating the results of these subimages using the dynamic time warping (DTW) algorithm [48]. Finally, to evaluate the performance of the DEKR-SPrior model on the entire soybean plant, we used a new test image dataset of whole soybean plants. This dataset comprises a comprehensive collection of high-resolution images of soybean plants captured under controlled conditions to ensure consistency and quality. Each image is carefully annotated to facilitate the precise localization and identification of individual soybean pods and seeds.

The main outcomes of the present investigation are as follows:

1. The DEKR-SPrior model, which is an extension of the DEKR framework, was developed. This model was introduced to address the challenges in soybean pod phenotyping. This model provides structural prior knowledge to improve keypoint clustering and pod discrimination, which are particularly useful for densely packed and overlapped pods.
2. A novel SPrior network was introduced to train soybean pods. This network incorporates cosine similarity to enhance the discrimination power of feature representation, which considerably boosts the accuracy of the model.
3. We demonstrated a strategy for increasing the resolution of the pod region in input images by cropping full-sized images into subimages, which enhances the model’s ability to identify pods with higher precision.
4. An additional test set comprising a high-resolution image dataset of whole soybean plants was generated to evaluate the performance of the DEKR-SPrior model on entire plants.
5. Our DEKR-SPrior model outperforms the previously developed bottom-up models, viz., Lightweight-OpenPose, OpenPose, HigherHRNet, and the original DEKR, on multiple pod keypoint detection, achieving a Pearson correlation coefficient (PCC) of 0.888 for pod counting and localization.

## Materials and Methods

### RGB image acquisition

The soybean plants were harvested from the experimental field of the Northeast Institute of Geography and Agroecology, Chinese Academy of Agricultural Sciences in Changchun, Jilin Province, China (43°88′N, 125°35′E), in 2020. The leaves and any debris associated with the soybean plants were removed, and the plants were subsequently placed into our custom-made data collection device for image capture (Fig. S1).

The image acquisition instrument consisted of an industrial camera, camera light, and white velvet background (Fig. S1). During image acquisition, a fixed white light source was used for illumination. A white velvet background cloth was used to reduce background interference. The detailed device information and an image of the device are shown in Fig. S1. The main process of image acquisition is described as follows: the operator inserts the plant into the socket and puts it on the ground. After the image of one soybean plant is taken, the operator pulls out the first plant and replaces it with a new plant for imaging. The resolution of the images taken is 2,048 × 3,000 pixels by default.

### Seed-to-pod annotation

**Fig. 1.**
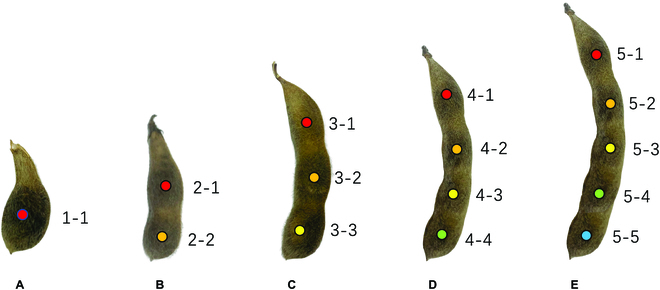
Different morphologies and annotations of pods. (A) 1-seeded pod. (B) 2-seeded pod. (C) 3-seeded pod. (D) 4-seeded pod. (E) 5-seeded pod.

We used the “Labelme image annotation tool” to annotate the data from 425 original full-sized images [49]. Each soybean seed is marked with a positional dot and given a label value (Fig. 1); the label value of each dot includes the following information: (a) the serial number uniquely assigned to each pod for identification, (b) the total number of seeds counted within the pod, and (3) the sequence number of the current seed being marked. The seed closest to the base of the pod is marked as the first seed in the sequence, while the remaining seeds are numbered in sequential order from the base toward the tip of the pod. This pod label is a unique identifier (ID) designated for every pod, and it ensures that each pod is distinct and identifiable among other pods. This unique ID constitutes the pod’s label sequence, which assists in precisely differentiating and identifying individual pods within the dataset (Fig. 1).

Here is an example illustrating the annotation process: suppose we have marked the position of the second seed in a 3-seeded pod with coordinates (*x*, *y*); then, its label would be denoted as “3-2(58)”, where the number “58” in the parentheses indicates that the ID of this particular 3-seeded pod is “58”. “3” represents the number of seeds in the pod, signifying that the current pod contains 3 seeds, and “2” is the sequence number of the seed currently being marked. Figure 1 depicts the varying morphologies of the pods and the corresponding annotations.

### Data preprocessing

In the current investigation, we used 2 different datasets to develop and validate our DL model. The first dataset comprises high-resolution subimages derived from full-sized images; this dataset is suitable for developing and validating the DEKR-SPrior model. The second dataset consists of full-sized images, which are exclusively utilized to test pod counting for entire soybean plants. Below, we have provided comprehensive details regarding these datasets along with the specific preprocessing steps adopted in this study.

#### High-resolution subimage dataset

High-resolution images allow the extraction of phenotypic features more precisely, thus providing accurate results. Prior to model input, we enhanced the resolution of the images by cropping the full-sized images into high-resolution subimages. We randomly selected 258 images from the 425 full-sized image datasets and manually calibrated the width of the soybean plant within each image. It was found that 90% of the soybean plants had widths within 400 pixels. Hence, we used a cropping size of 400×400 pixels (as illustrated in Fig. S2) and adjusted the annotations that corresponded to the new coordinates. Considering the prevalence of blank spaces in the images, cropping with a fixed stride might result in subimages that are devoid of any pods. To ensure that the subimages contained as many pods as possible, our selection was based on the annotated information, and one pod was randomly chosen each time and expanded from the minimum bounding rectangle of the current pod in all 4 directions—up, down, left, and right—to obtain a subimage 400×400 pixels in size.

However, to avoid the overlapping of images between the training and testing sets, i.e., the same pods should not be represented in the training and testing sets, we initially divided the 258 images into training and testing sets before cropping the images. This process resulted in 3,700 subimages for training, which included 32,780 pods and 78,920 seeds. In addition, 205 subimages were utilized for testing, including 1,856 pods and 4,520 seeds. A statistical analysis of this dataset is provided in Table 1 and Fig. S3.

**Table 1.** The descriptive statistics of the dataset include the high-resolution subimage dataset and full-sized image dataset. #Image stands for the number of images. Avg #Pod and Avg #Seed denote the average number of pods and seeds per image, respectively. *N*-seeded Pods denotes the total number of *N*-seeded pods.

Datasets	High-resolution subimages dataset		Full-sized images dataset
	Train	Test	Test
#Image	3,700	205	167
Total #Pods	32,780	1,856	8,426
Total #Seeds	78,920	4,520	22,071
Avg #Pod	8.86	9.05	50.46
Avg #Seed	21.33	22.05	132.16
1-seeded #Pods	6,462	372	1,035
2-seeded #Pods	9,778	515	2,469
3-seeded #Pods	13,266	758	3,610
4-seeded #Pods	3,266	211	1,292
5-seeded #Pods	8	0	20

#### Full-sized image dataset

The high-resolution subimages dataset is predominantly utilized to train models with high precision; hence, the above subimages dataset was also utilized as a training set in this case. Moreover, the full-sized image dataset assists in quantifying the total number of pods on an entire soybean plant, thereby assessing the performance of the model in recognizing the entire plant. The image dataset that remained uncropped consisted of 167 annotated images and was distinct. The statistical characteristics of this dataset are delineated in Table 1 and Fig. S4.

### DL framework for pod detection and localization

The keypoints of the soybean pod were drawn by using a concept that is analogous to the human body. We proposed that a soybean pod can be viewed as a human body with up to 5 distinct body parts. This framework allows us to adapt bottom-up models used in human pose estimation for the detection of keypoints on soybean seeds. In this analogous concept, each pod is considered an individual, and each seed within the pod represents a joint. The structure of the soybean pod resembles a simple chain consisting of 5 joints without any branching.

Examples of pods containing 1 to 5 seeds illustrate this concept (Fig. 1). Generally, the number of seeds per pod ranges from 1 to 4, and pods with more than 5 seeds are exceptionally rare. Hence, we assume that a single soybean pod possesses a maximum of 5 seeds. Under this assumption, each pod is treated as an entity with up to 5 distinguishable parts. However, if the pod contains fewer than 5 seeds, we consider that the missing seeds are sealed. For example, a pod with a single seed can be considered a person with only their first body part visible, while the subsequent parts from 2 to 5 are sealed. The same concept can be used to interpret the structure of pod types with varying seed numbers.

**Fig. 2.**
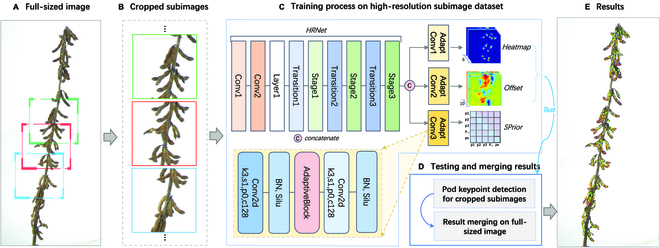
Research flow diagram. (A) Original full-sized image. (B) Subimages obtained through cropping. (C) Network for training and testing. (D) Full-sized image testing and result merging. (E) Results depicted on the full-sized image.

An outline of the proposed method and an illustration of the system workflow are presented in Fig. 2. The process begins by using the colored images as input and culminates in the generation of exact 2-dimensional (2-D) positional data for the anatomical keypoints of each pod present in the image. Many full-sized images of 258 different soybean plants were collected using a specialized apparatus, as shown in Fig. S1. These images are carefully labeled and cropped to generate high-resolution subimages, which are subsequently used as the input dataset for training and validation of the bottom-up models.

In the current investigation, we used the DEKR-SPrior model, which functions through 3 distinct but parallel branches, viz., the heatmap branch, the offset branch, and the innovative SPrior module. Each branch possesses a specific function, and a specialized adaptive convolution network is utilized to accurately detect and determine the position of the respective seeds.

In addition to the cropped subimages used in the training phase, we also used a full-sized image dataset of 167 different soybean plants for testing. This assessment requires a careful count of all pods present across the entire soybean plant, as extracted from the original full-sized image. Initially, we cropped the full-sized images into subimages. The DEKR-SPrior model is then used to determine the positional data of the pods within these cropped subimages, which relies on the heatmap and offset outputs. Following this evaluation, the results obtained were subjected to rigorous merging and filtering processes. This phase is very important because it enables the development of a detailed phenotypic profile for mature soybean plants that includes information on pod type, total pod count, and seed count. This comprehensive analysis is crucial for obtaining in-depth information on plant phenotypic traits.

### Model architecture and training process on a high-resolution subimage dataset

The backbone of DEKR-SPrior is HRNet, which trains on high-resolution representations of input data to perform recognition tasks. After the last feature extraction layer, stage 3, the original DEKR is divided into 2 branches, namely, a heatmap, which is used to generate the confidence map of the seed keypoints, and an offset branch, which is used to locate the positions of the seeds. The confidence map is a 2-D representation of the assumption that a particular pod seed occurs at each pixel location. The pixelwise keypoint regression framework estimates a candidate soybean pod at each pixel by predicting the 2*K*-dimensional offset vector and *K*-dimensional confidence vector from the center pixel for the *K* keypoints. Our study is based on soybean pod detection and localization; hence, there are 6 heatmaps (one as background) and 10 offsets for regressing the 5 keypoints.

The DEKR-SPrior model incorporates an additional branch known as the SPrior module, which can extract features from pod images via a convolutional neural network (CNN). This module can capture the channel features of all the seeds presented in the feature maps. A distinct convolutional layer known as “Adapt Conv3”, highlighted within a yellow background, is employed to isolate structural feature maps. By considering the basic CNN architecture, which comprises convolutional layers, batch normalization (BN) layers, and SiLU activation layers, the SPrior module also integrates adaptive convolution. This extra module facilitates the learning of relationships between proximate keypoints and enables the model to develop a focused representation within the keypoint region, thereby refining the feature extraction process for pod recognition tasks.

DEKR uses average precision (AP), which is a main competition metric in common objects in context (COCO) to person keypoint challenges and is a primary metric for evaluating model performance. In comparison to human pose estimation, soybean pod detection estimation has many challenges, as discussed below:

1. The main factor affecting the area of the bounding box is not only the scale but also the number of seeds in the pod, as shown in Fig. S5.
2. In contrast to those in human body parts, the labels of seeds in the same pod are generally problematic.
3. The differences in image features between different seed types (such as between the first seed and second seed) are relatively unsubstantial compared with human body parts (e.g., between an elbow and a knee).

Because of the 3 abovementioned challenges, the original object keypoint similarity (OKS) metric within the AP is insufficient to fully represent the actual performance of the model. In the “Improved OKS in AP” section, we introduce modifications to the OKS metric that are better suited to the specific challenges of soybean pod detection.

The performance of our model was evaluated using the AP with the improved OKS metric based on 205 test subimages.

#### Structural prior module

**Fig. 3.**
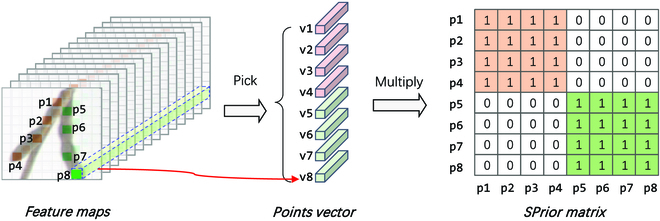
Diagram illustrating the structural prior module of the DEKR-SPrior model.

The SPrior module is a “novel addition” and is designed to measure the relationships between seeds, thereby ascertaining whether the detected seeds belong to the same pod. As shown in Fig. 3, the SPrior module comprises point vectors and an SPrior matrix. The SPrior module uses the feature maps along with the positional data of the seed keypoints as input and provides a matrix as an output that represents the correlations between these keypoints. Notably, the SPrior module is exclusively utilized in the training phase and does not participate in the prediction phase; hence, it does not influence the potential of the model’s inference.

**Points vector.** Before calculating the affinity matrix of keypoints, the feature vector of each keypoint must be obtained as the structural feature. Suppose that a total of N seeds are in an image. For each keypoint, we obtain the feature across all channels at its position in the feature maps $F\in R^{H\times W\times C}$, where *H*, *W*, and *C* are the height, width and channel of the feature maps, respectively. These feature maps form a vector of dimension ***v***_***i***_ ∈ *R*^1×1×*C*^, ∀*i* = 1, …, *N*, followed by flattening to obtain the point vector of length *C*. The association matrix ***A*** ∈ ***R***^*N*×*N*^ between keypoints ***P*** = {*p_i_* | *i* = 1, 2, …, *N*} can be obtained by calculating the cosine similarity of the vectors:

$$A_{i,j}=\left(v_{i}^{T}∙v_{j}\right)/\left(\left\|v_{i}\|\|v_{j}\right\|\right)$$ (1)

**SPrior matrix.** We argue that the degree of association between seeds belonging to the same pod is greater than that between seeds belonging to different pods. We consider each seed as a node, denoted as *p_i_*, *i* ∈ {1, 2, …, *N*}, and the seeds belonging to the same pod are connected. Therefore, a matrix ***A′*** ***∈ R***^*N* × *N*^, similar to the adjacency matrix in the field of knowledge graphs, can be constructed. We call ***A′*** the SPrior matrix. If *p_i_* and *p_j_* belong to the same pod, $A_{i,j}^{\prime }=1$; otherwise, $A_{i,j}^{\prime }=0$.

**Loss function of SPrior.** In the SPrior module, we hope that the matrix ***A*** calculated by the points vector is as close as possible to ***A*^′^** obtained by the true value of the seed keypoint. Therefore, the SPrior loss is defined as the L2 loss between ***A′*** and ***A***:

$$L_{\text{SPrior}}={\left\|A-A^{\prime }\right\|}_{2}^{2}$$ (2)

**Optimization loss.** DEKR uses a multibranch network to learn disentangled representations for keypoint regression. The loss includes heatmap loss and keypoint regression loss:

$$L_{\text{original}}^{\text{dekr}}=l_{\text{heatmaps}}+{\gamma l}_{p}$$ (3)

where *l_heatmaps_* is the entrywise 2-norm of weighted distances between the predicted heat values and the ground-truth heat values, *l_p_* is a normalized smooth loss for pixelwise keypoint regression, and *γ* is a trade-off weight.

In our method, the whole loss is defined as the sum of the heatmap loss $L_{\text{original}}^{\text{dekr}}$ and the SPrior loss, where *ρ* is a hyperparameter for balancing 2 losses:

$$L=L_{\text{original}}^{\text{dekr}}+{\rho L}_{\text{SPrior}}$$ (4)

#### Improved OKS in AP

The AP computes the average precision value for recall values of 0 to 1, and a high AP indicates a stable and consistent model across different confidence thresholds. The AP value also equals the area under the precision–recall (PR) curve. The PR curve shows the trade-off between the precision and recall values for different thresholds. This curve helps to select the best threshold to maximize both metrics.

The AP metric is defined according to the OKS, which can evaluate the similarity between the predicted and ground-truth keypoints. It is calculated from the distance between the predicted points and ground-truth points normalized by the scale of the object. The original OKS is calculated as follows:

$$\mathrm{OKS}=\left(\sum_{i}\text{exp}\left(-\frac{d_{i}^{2}}{2s^{2}k_{i}^{2}}\right)\delta \left(v_{i}>0\right)\right)/\sum_{i},\delta ,\left(v_{i}>0\right)$$ (5)

In Eq. 5, *d_i_* is the Euclidean distance between the ground truth and the detected keypoint for the *i*th part, *v_i_* is the visibility flag of the ground truth, *s* is the square root of the ground-truth bounding box area, and *k_i_* is a per-keypoint constant that controls falloff. We see that *s* × *k_i_* is the standard deviation of this Gaussian distribution. In practice, *k_i_* = 2*σ_i_* represents the variance of deviation caused by the different labeling difficulties. *σ_i_* can be calculated as follows:

$${\sigma_{i}}^{2}=E\left[d_{i}^{2}/s^{2}\right]$$ (6)

We note that the number of seeds in the pod is an important factor in determining the area of the bounding box, in addition to the scale. For the OKS metric to focus on the difference in the area of the bounding box caused by the scale rather than the number of seeds, an area correction term is needed.

We propose an improved evaluation metric based on *OKS* that better suits our situation:

$${\mathrm{OKS}}_{\mathrm{pod}}=\frac{1}{n}\sum_{i=1}^{n}\exp\left(-d_{i}^{2}/\left(2\lambda_{n}s^{2}k^{2}\right)\right)$$ (7)

In Eq. 7, the terms with visibility flags presented in Eq. 5 are omitted since they do not exist in our dataset. *n* is the number of seeds in the pod. *λ_n_* is an area-correction coefficient for pods with *n* seeds, which depends on the bounding box size ratio of different pod types. This approach enables us to eliminate the effect of the different bounding box areas caused by different numbers of seeds in the pods. *k* is a constant that is no longer related to the seed type and is calculated as *k* = 2*σ*.

We assumed that all seed types had identical labeling difficulties. To select a proper value for *σ*, we first pick up 10 images from the labeled dataset and carefully relabel the pod positions to achieve a more precise ground truth. Then, we calculate the mean distance deviation between the refined and original annotated positions. We finally obtain the constant value of *σ* via Eq. 6.

For *λ_n_*, we pick 10 images at random and calculate the average ratio between the bounding box area of 3-seeded pods and that of *n*-seeded pods in the same image. The values of *λ_n_* are set as [4.752, 1.353, 1, 0.782, 0.673] for *n* = 1, 2, 3, 4, and 5, respectively.

If pods with different numbers of seeds have similar target scales, their *OKS_pod_* values should only depend on the mean distance between the predicted and ground-truth positions. In other words, the Gaussian curves for pods with different numbers of seeds have the same standard deviation (*s* × *k_i_* ), as shown in Fig. S6.

### Testing and the results obtained from merging on a full-sized image dataset

The trained model obtains the pod detection results only from cropped subimages. However, the performance of this trained model is tested in terms of pod detection and localization in the original full-sized images that are obtained directly without cropping as well as the cropped subimages.

In this process, we crop the image based on a fixed stride and record the coordinates of each subimage within the original image, thereby enabling us to trace the pods from the subimages back to their locations in the original image. We first integrate the detection results of all the cropped subimages, filter out some closely spaced pod seeds through Euclidean distance calculations, obtain the seed keypoints of the detected pod results, and record the position sets *LOC* of the subimages, which are *x_min_*, *x_max_*, *y_min_*, and *y_max_*. Then, for each *LOC*, we obtain the expanded areas *M_left_*, *M_right_*, *M_top_*, and *M_down_* of the cropped area in 4 directions, and the soybean pods in these areas are obtained. These 4 areas refer to the regions that we have cropped and that need to be merged.

In these 4 regions, the pods that typically require merging do not necessarily coincide in position but share a high degree of similarity in their morphological distribution. This attribute essentially translates to an alignment or matching challenge between 2 sequences of varying lengths. The DTW algorithm provides an effective solution by allowing for local stretching within sequences to identify the optimal (minimum distance) alignment or match, thereby addressing our challenge. Specifically, we first calculate the Euclidean distance *dist_norm_seeds* between all seed keypoints in the area and retain all seed pairs smaller than the specified threshold *dist_thresh*. If the 2 seeds do not belong to the same pod, we must proceed as follows: if the pod has only one seed, then the 2 pods are merged directly; if not, the DTW algorithm is used to calculate the morphological similarity *shape_dist* of the 2 pods. If *shape_dist* is less than the specified threshold *shape_thresh*, the 2 pods are merged, and the connection relationship calculated by DTW is the merged combination. The main flow to merge the results is shown in Algorithm 1. Model performance was evaluated using the mean absolute error (MAE) based on 167 full-sized testing images.


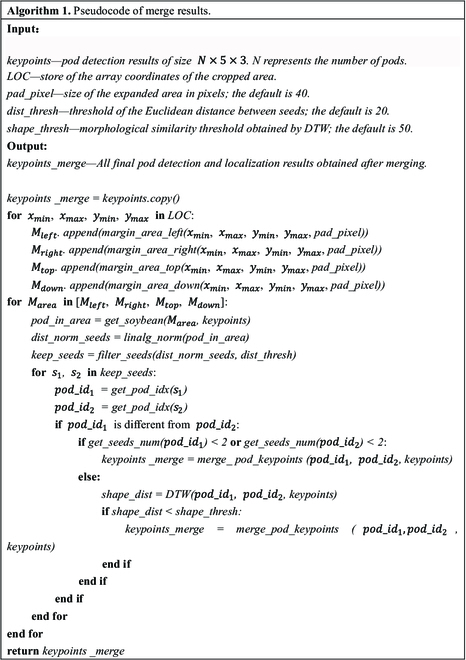


### Experimental setting

The experiment was performed on the PyTorch framework with a GPU (a server with 8×NVIDIA A100 GPUs). To maintain the integrity of the pods during training, we use only flipping for data augmentation. For experimental comparison, we also conducted experiments using previously classical bottom-up models, viz., Lightweight-OpenPose [35], OpenPose [39], HigherHRNet [37], and the original DEKR [38].

For Lightweight-OpenPose, the hyperparameters are as follows: a learning rate of 1e-4, an input image base height equal to 368 pixels, and 300 epochs for model training. Testing was performed without multiscale input using a heatmap threshold of 0.2, a pixel-level Euclidean distance threshold of 6 pixels between soybean seeds, and a minimum PAF threshold of 0.05.

The OpenPose parameters include the CPU pose framework with the initial convolutional network layers derived from VGG-19, which are fine-tuned thereafter. The learning rate is 0.1 with 300 epochs. The test utilized a heatmap threshold of 0.1, a PAF threshold of 0.05, and an NMS threshold of 0.4.

The HigherHRNet configurations consist of an HRNet-w32 backbone, an image input size of 512 pixels, 2 stages, an “exp” type for AE loss, and 400 epochs. The heatmap threshold was set to 0.1.

For DEKR, the backbone is HRNet-w32, with a trade-off weight *γ* in Eq. 3 of 0.03 and 400 epochs for training. The testing phase applied an NMS threshold of 0.05 and a heatmap threshold of 0.3.

The DEKR-SPrior model adheres to the same settings as DEKR, with ablation studies conducted on the SPrior loss parameter ρ in Eq. 4 to assess its influence on the outcome performance.

Across all the models, the Adam optimizer is utilized with a batch size of 90.

## Results

### Comparison of the performances of the DEKR-SPrior model and other bottom-up models on a high-resolution subimage dataset

**Fig. 4.**
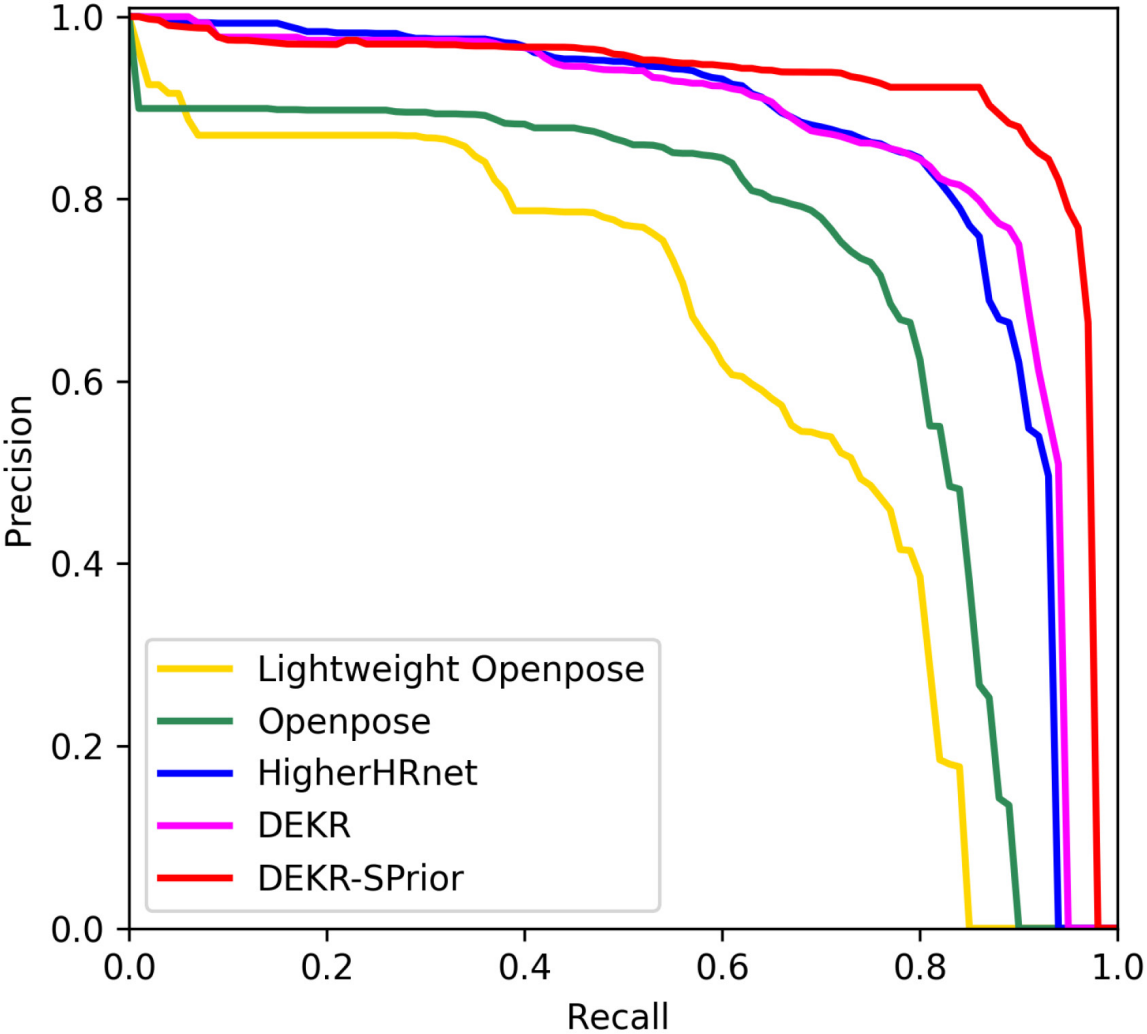
PR curves for different bottom-up models, namely, Lightweight-OpenPose, OpenPose, HigherHRNet, DEKR, and DEKR-SPrior.

In this study, we compared the performance of our model, viz., DEKR-SPrior, with 4 previously reported bottom-up models, viz., Lightweight-OpenPace, OpenPose, HigherHRNet, and the original DEKR (Table 2**)**. We analyzed the results of our model and the 4 previous models on our test set, viz., 205 cropped images from soybean plants. The performances of the 5 models are presented in Table 2. Our results showed that our model, i.e., DEKR-SPrior, had AP, AP_50_, AP(1-seeded), AP(2-seeded), AP(3-seeded), and AP(4-seeded) values of 72.4%, 91.4%, 71.7%, 80.9%, 85.6%, and 83.6%, respectively (Table 2**)**. Compared to the DEKR model, DEKR-SPrior showed improvements of 1.1%, 4.4%, 5.2%, 3.6%, 2.3%, and 3.6% in the AP, AP_50_, AP(1-seeded), AP(2-seeded), AP(3-seeded), and AP(4-seeded), respectively. DEKR-SPrior achieved the best result for soybean pod keypoint detection, and the overall AP of DEKR-SPrior was 29.6% greater than that of Lightweight-OpenPose. After adding the SPrior module to DEKR, the AP values of the 2-seeded and 3-seeded pods increased substantially (Table 2**)**. This result proves that the SPrior module effectively improves the accuracy of the DEKR-SPrior model. Moreover, the PR curves showed that the precision of the other methods, viz., Lightweight-OpenPose, OpenPose, HigherHRNet, and DEKR, decreased markedly as the recall increased (Fig. 4). The PR curve of DEKR-SPrior is more inclined to the upper right corner than that of Lightweight-OpenPace, OpenPose, HigherHRNet, and DEKR. Hence, these results showed that for a given recall rate, DEKR-SPrior can maintain high precision and solve the problems of missed detection and incorrect detection more effectively.

**Table 2.** Results of the subimages testing dataset, where DEKR-SPrior demonstrates the best performance compared to other models

Model	AP	AP_50_	AP(1-seeded)	AP(2-seeded)	AP(3-seeded)	AP(4-seeded)
Lightweight-OpenPose	45.7	68.9	12.5	52.9	60.4	58.4
OpenPose	58.6	82.3	43.0	54.6	66.0	60.9
HigherHRNet	71.6	89.2	68.0	78.3	83.1	81.4
DEKR	71.3	87.0	66.5	77.3	83.3	80.0
DEKR-SPrior	72.4	91.4	71.7	80.9	85.6	83.6

**Fig. 5.**
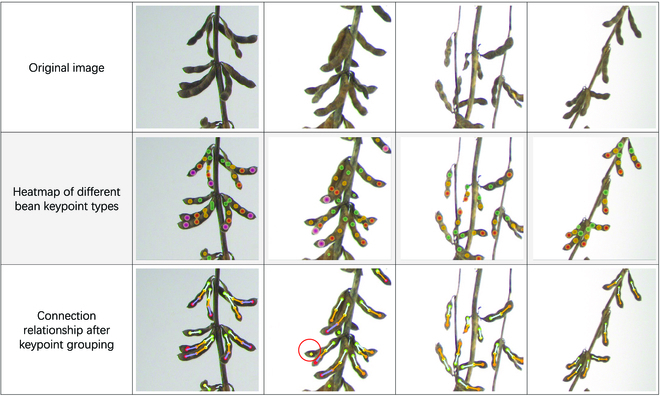
Visualization of the results in the subimage testing dataset.

In addition, some of the pod detection results are shown in Fig. 5. The first row shows the cropped images, which are the original image input dataset for the DEKR-SPrior model. The second row shows the heatmaps of different seed/keypoint types. Each color represents a specific seed type; for example, green, yellow, orange, and pink represent the first, second, third, and fourth seed types of the pod, respectively. The more distinct the color is in the heatmap, the more accurately the location of the seed type belonging to the particular pod is recognized. The figure clearly shows that the model correctly finds the location of all seed types irrespective of how the pod is placed, i.e., facing up or down (see the second row of Fig. 5). The third row is the connection relationships among the seed types after the keypoint groupings are derived from the second row. After finding the positions of all the seeds from the heatmap in the second row, the AE algorithm is used to aggregate the connection relationships among all the seeds. In particular, the first seed type can only be connected to the second seed type, as shown by the cyan line, the second seed type can only be connected to the third seed type, as shown by the orange line, and so on, until connecting to the fifth seed type. As shown in Fig. 5, the positions of different seeds belonging to the same pod are correctly identified and connected in order according to the seed type. Even if 2 pods are very close to each other, they can be correctly identified, and the positions of their respective seeds can be accurately identified (as shown by the red circle in the second picture). When the pod is obscured, the type of pod can also be determined according to the visible part.

In our study, we performed ablation analyses on the loss function and the SPrior module, and the results are presented in Fig. S7. The hyperparameter *ρ* signifies the extent of SPrior’s contribution to the model’s computations, with larger values indicating greater influence. Our results demonstrated that incorporating the SPrior module enhanced the model’s performance. Specifically, when *ρ* was set to 0.01, the AP improved from 71.30% (original DEKR) to 71.96% (in DEKR-SPrior) (Fig. S7). Our results revealed that the accuracy of the model increased notably as the SPrior weight increased, with the AP value progressively increasing until it peaked at 72.43% when *ρ* = 0.2 (Fig. S7). Beyond this point, AP decreased with a further increase in the weight, suggesting that *ρ* has a particular threshold at which the maximum value of AP is obtained; hence, there is a need to fit this threshold value in the training dataset. In other words, this trend indicates that while the SPrior module considerably benefits the model by enhancing the discriminability of features, there is an optimal balance for *ρ*. Exceeding this threshold could make the model more sensitive to the structural cues provided by the SPrior, leading to the loss of the benefits provided by the SPrior.

Hence, the results of this study revealed the need to carefully tune *ρ* to the threshold value in the training dataset. This optimization is essential for harnessing the true potential of the SPrior module and ensuring that the model can be trained from the training dataset to increase the precision of the testing dataset. Our results indicate that the AP initially improved and then decreased with increasing *ρ*, suggesting the existence of an optimal *ρ* value that yields the best performance according to the DEKR-SPrior model. To identify this optimal value and prevent overfitting, further research is required to thoroughly investigate the specific impact of varying *ρ* on model efficacy. Additionally, refining the integration strategy of the SPrior module is necessary to achieve consistent and stable performance enhancements across diverse datasets.

### Comparison of detection effects on a full-sized image dataset

The Pearson correlation coefficient (PCC) and MAE for seeds and pods from our full-sized image dataset are presented in Table 3. The MAEs of seed counting for the Lightweight-OpenPose, OpenPose, HigherHRNet, DEKR, and DEKR-SPrior models were 33.32, 30.68, 27.37, 25.81, and 21.11, respectively, and the PCCs of the Lightweight-OpenPose, OpenPose, HigherHRNet, DEKR, and DEKR-SPrior models reached 0.701, 0.744, 0.815, 0.852, and 0.881, respectively. In the case of pod counting, DEKR-SPrior performs considerably better than the original DEKR, with an MAE and a PCC equal to 5.29 and 0.888, respectively. Moreover, DEKR-SPrior has the best overall performance in terms of seed and pod counting among all the other studied bottom-up models, viz., Lightweight-OpenPose, OpenPose, HigherHRNet, and DEKR.

**Table 3.** Results of the full-sized image dataset. DEKR-SPrior demonstrates the best performance compared to other models in both MAE and PCC metrics.

Methods	Seeds		Pods	
	MAE	PCC	MAE	PCC
Lightweight-OpenPose	33.32	0.701	10.82	0.713
OpenPose	30.68	0.744	9.41	0.772
HigherHRNet	27.37	0.815	6.92	0.829
DEKR	25.81	0.852	6.06	0.836
DEKR-SPrior	21.11	0.881	5.29	0.888

**Fig. 6.**
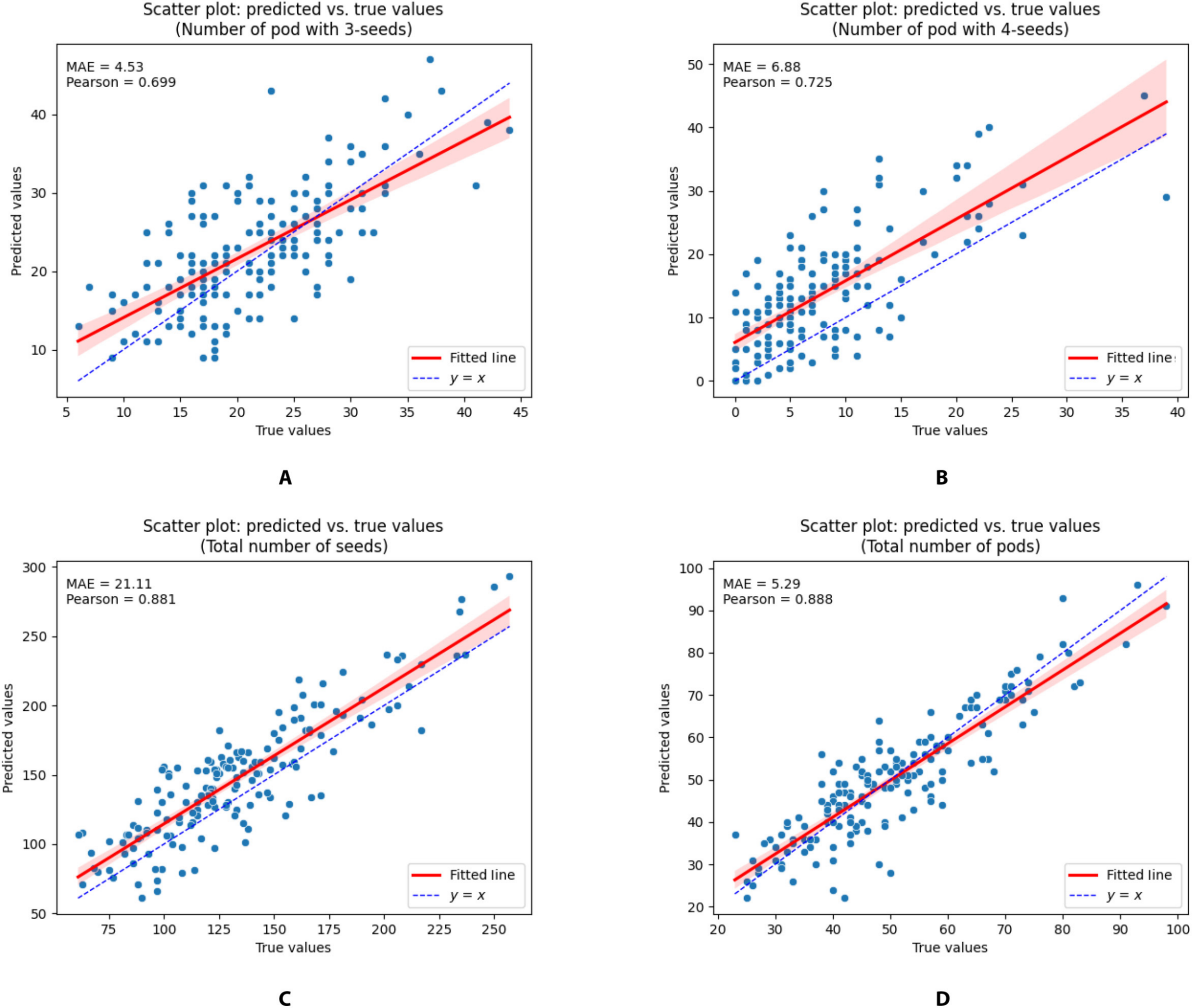
Scatter plot and fitted curve of true values vs. prediction values. (A) The number of 3-seeded pods. (B) The number of 4-seeded pods. (C) The total number of seeds. (D) The total number of pods.

Scatter plots and fitted curves of true values vs. prediction values are provided in Fig. 6. The blue dashed line represents the ideal scenario where true values and predicted values perfectly align. The red solid line represents the fitted curve. The numbers of 3-seeded and 4-seeded pods are presented in Fig. 6A and B, respectively. The 3-seeded pods are obviously more numerous and have lower MAE values than the 4-seeded pods; hence, the larger the amount of data used for model training is, the better and more accurate the results obtained. The results of the total number of seeds and pods are presented in Fig. 6C and D, respectively. The model provided excellent phenotypic results for identifying the total number of pods and the total number of seeds, especially the PCC for the total number of pods, which reached 0.888. Hence, DEKR-SPrior had excellent performance in pod detection at the maturity stage.

## Discussion

Conventional methods for soybean pod phenotyping are inefficient, expensive, prone to errors, and imprecise. Recently, there has been a dramatic shift toward the use of high-throughput image-based phenotyping techniques for detecting seeds and pods [50]. Visible images allow the automatic recognition of the color and texture of an object, thus offering a cost-effective and rapid analysis method [51]. Progress has recently been made in estimating human poses based on images using different methods, especially the DEKR method [38,43,52]. We believe that the DEKR method can accurately identify the keypoints necessary for labeling pods during model training; thus, this method is expected to more precisely locate and count soybean pods and seeds. However, applying the DEKR model directly will provide suboptimal performance, primarily because the original DEKR was designed to accommodate various human body postures rather than to recognize soybean pods. For increased performance and efficient use in the location and counting of soybean pods and seeds, we added SPrior to the original DEKR model, resulting in the development of the DEKR-SPrior model. This upgraded model is used for soybean pod detection and counting and offers improved accuracy, especially when pods are densely packed and overlapped.

In comparison to the human body, which possesses considerable differences among body parts, the color and morphology of each seed in the soybean pod are extremely similar, which affects the accuracy of the deep neural network in pod detection. The bottom-up models focus on studying the geometric constraints and structural relationships among the keypoints; these models first identify the location of the seeds in each pod from the image, and then, they group the seeds based on the pods. In DEKR, it is believed that to accurately identify the location of the pods in the image, it is necessary to focus on the region where the pods are located and learn the location of the keypoints from the seed region. The DEKR model uses adaptive convolution to activate the pixels in the region where the keypoints are located so that it can focus on the region where each keypoint is located. Moreover, a method for separating regression between keypoints is adopted, i.e., through a multibranching structure, where each branch learns one type of keypoint representation and the branch has an adaptive convolution dedicated to that keypoint, thus effectively improving the recognition progress. Our results revealed that compared to the other bottom-up models, the original DEKR model possesses more obvious advantages in pod detection (Tables 2 and 3). However, unlike the flexible posture of the human body, soybean pods are relatively fixed, the connecting line between several soybean seeds can be considered an approximately straight line, and the original DEKR has not imposed restrictions on the recognition results. Our proposed DEKR-SPrior model enhances the positional structural constraints between pods by incorporating a parallel independent branch. This addition allows the DL model to learn a more precise feature representation for each pod. In the case of multipod pose estimation, feature clustering is a critical step that combines detected seeds into pods. Our proposed SPrior module enhances the ability of the DEKR-SPrior model to distinguish between different seed features by incorporating cosine similarity, which improves the clustering process of seed features. This improvement, in turn, enhances the ability of the DEKR-SPrior model to make the grouping of seeds more accurate for the same soybean pod. Thus, the DEKR-SPrior model reduces the occurrence of incorrect connections and more accurately links the seed keypoints, thereby improving the accuracy of pod recognition. Additionally, the SPrior module enhances feature representation by introducing structural constraints posterior to the feature representation layer. It uses 2 layers, a convolution layer with a SiLu-based activation function and an adaptive convolution layer. The adaptive convolution uses the affine transformation to generate offsets for each feature point; this action is followed by deformable convolution, which adaptively extracts keypoint features based on the computed offsets. This action ensures that the extracted feature points are concentrated near the keypoints. In the case of pod recognition, which overlaps with each other as well as closely positioned pods, the SPrior module considerably enhances the feature representation within the pods, thereby enhancing the DEKR-SPrior model recognition accuracy.

The models, viz., Lightweight-OpenPose and OpenPose, use PAF, and HigherHRNet and DEKR use AE [53]. The modeling results presented in Table 2 clearly show that the AE clustering method is more suitable for pod recognition. PAFs possess information about the position and orientation of each pod, where each pixel describes a 2-D vector [39]. In contrast, the AE learns an embedding representation for each pod [54]. By comparing the tag values in the tap map corresponding to the peak position of the seeds in the output, seeds with similar tag values are clustered into one pod. However, the AE does not pay special attention to the orientation relationship between the 2 seed points in front and behind and focuses more on learning its own features of the seeds in each pod [41]. The direct clustering method based on the learned feature representation is obviously more suitable for the task of pod identification. Similarly, we have added a direct clustering method for each seed in the SPrior module, which assigns enhanced category labels to the seeds belonging to the same pod so that close seeds have a greater probability of belonging to the same pod. After learning through this labeling enhancement, DEKR-SPrior exhibits superior results to all previous bottom-up models, viz., Lightweight-OpenPose, OpenPace, HigherHRNet, and the original DEKR.

The labeling of the seed keypoint position of each pod requires the information of the seed location in the pod and which pod the seed belongs to. In our study (Table 1), we reported that the average number of pods per soybean plant was 50.46, and the average number of seeds was 132.16 (Table 1). This assessment requires labeling not only for the locations of approximately 132 seeds but also for the individual labels of approximately 50 pods to which each seed belongs. This project is extremely time-consuming compared to the previous methods. However, DL-based methods are advantageous because the larger the amount of input data provided is, the greater the accuracy of the recognition. In our study, we labeled the images without cropping them and used them as input data directly into the bottom-up model for training. The limited amount of data we possess, i.e., only 258 full-sized images were utilized for training and validation, results in poor model convergence and thereby a loss of ability to accurately recognize the output results in the validation set. In contrast, when we trained the model by cropping all full-sized images into high-resolution subimages and increasing the number to 3,500, the bottom-up models achieved good convergence and had a greater ability to provide accurate output results in the validation set. Ultimately, in the case of the validation set involving the full-sized images, the MAEs for seeds and pods in the DEKR-SPrior model were 21.11 and 5.29, respectively. These ratios corresponded to 15.9% and 10.4% of the mean values, respectively, indicating that the DEKR-SPrior model has great potential for seed and pod recognition. Using the same amount of annotated data, we obtained superior results with the subimage method, which underscores the efficacy of this approach in identifying small targets within high-resolution images.

In the present study, we proposed an upgraded bottom-up keypoint detection method named DEKR-SPrior, which possesses greater ability and efficiency for soybean seed and pod localization, as well as seed and pod counting. DEKR-SPrior is much more accurate than previous bottom-up models, viz., Lightweight-OpenPose, OpenPace, HigherHRNet, and the original DEKR. DEKR-SPrior addresses the challenges of the previous bottom-up methods by accurately quantifying soybean pod characteristics, predicting seed keypoints, and grouping them according to distinct pods. Because of the close proximity and greater overlap of soybean pods, in situ pod detection and localization are considerably more difficult to identify than the pods removed from soybean plants. By cropping the full-sized image to high-resolution subimages and using the cropped images as a training set, the DEKR-SPrior model achieved excellent results for in situ plant location as well as for soybean pods and seeds enumeration. The SPrior module is used to enhance representation learning during the training phase. In addition, we propose an improved evaluation metric *OKS_pod_* based on *OKS* by adding an area correction term. We conducted extensive experiments on our self-collected image dataset by comparing the potential of DEKR-SPrior with that of multiple bottom-up models (Lightweight-OpenPace, OpenPose, HigherHRNet, and DEKR). Our results revealed that the SPrior module can substantially improve the AP value. The results demonstrate that the proposed DEKR-SPrior can mitigate the low efficiency and accuracy of soybean trait phenotyping. In conclusion, we developed a new bottom-up model, DEKR-SPrior, which is more efficient at phenotyping the pod number and seed number in soybean; therefore, this model can be used to accurately estimate soybean yields.

## Data Availability

The source code is publicly available. It can be accessed at the following GitHub repository: https://github.com/Cyncihe/DEKR-SPrior.git. In addition, some of the homemade datasets used in this study can be obtained by contacting the corresponding author.
