# Supplementary material for: DEKR-SPrior: An Efficient Bottom-Up Keypoint Detection Model for Accurate Pod Phenotyping in Soybean
Source: Plant Phenomics. 2024 Jun 27;6:0198. doi: 10.34133/plantphenomics.0198 (PMC11209727; doi:10.34133/plantphenomics.0198)
Supplement: Supplementary 1 — Figs. S1 to S7 [file plantphenomics.0198.f1.zip › Supplementary Materials-v2.docx]

**Supplementary Materials**


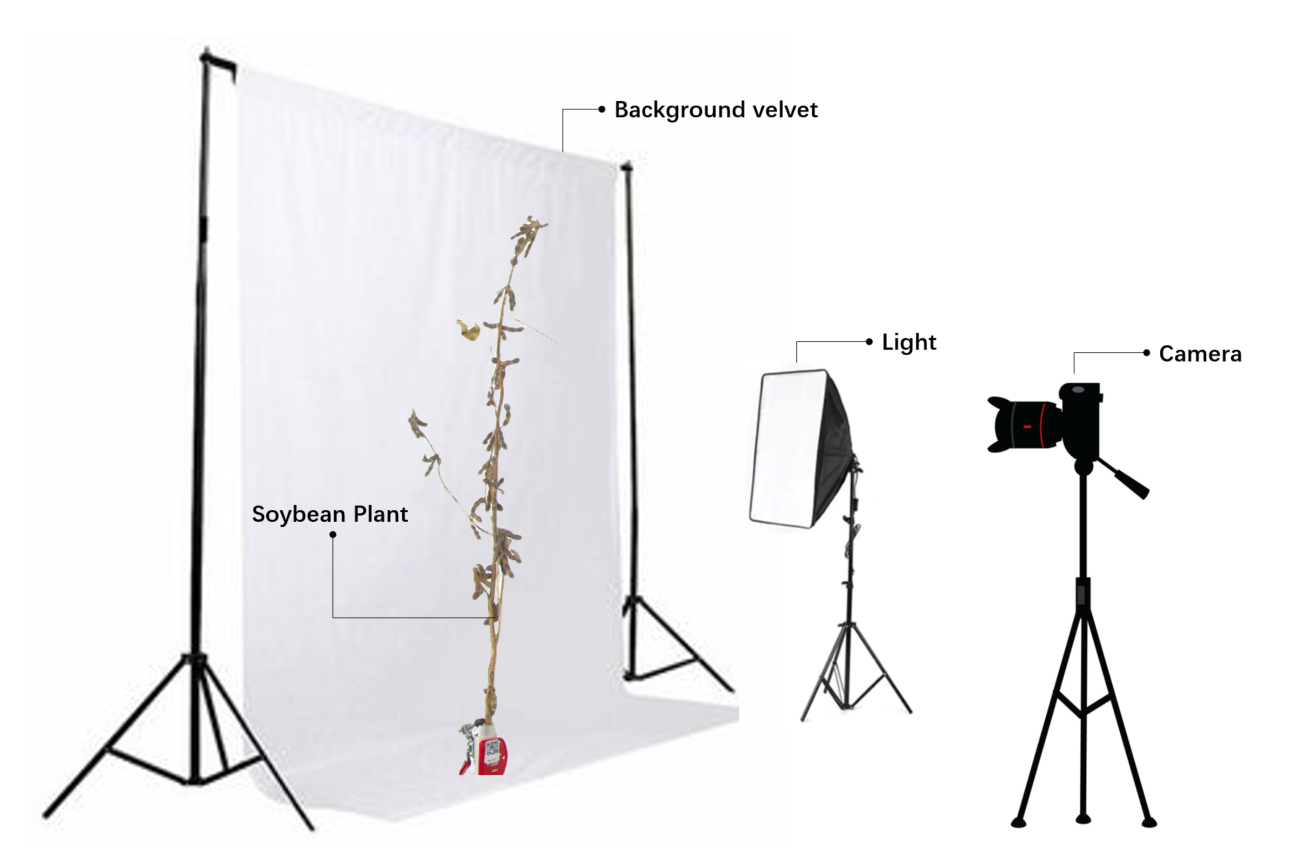


*Fig.S1. Image acquisition scene demonstration.* *The image acquisition instrument is composed of an industrial camera, camera light, and a photography background velvet.*


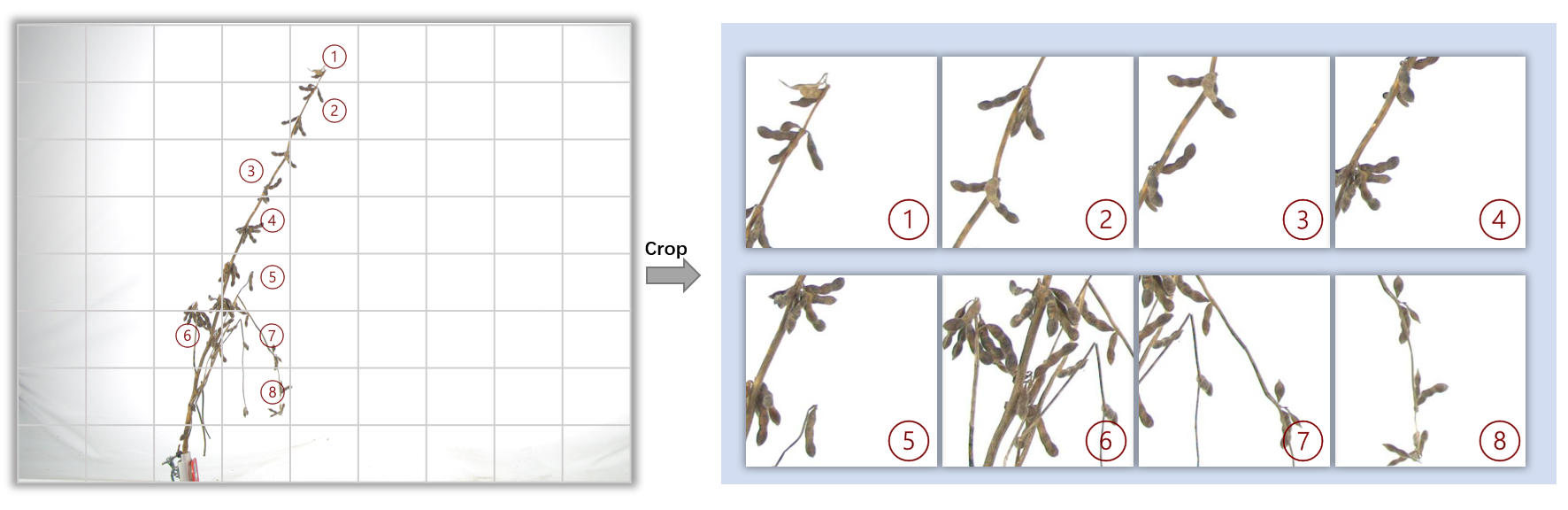
*Fig. S2. Images cropping process. The original full-size images are cropped into multiple sub-images.*

| *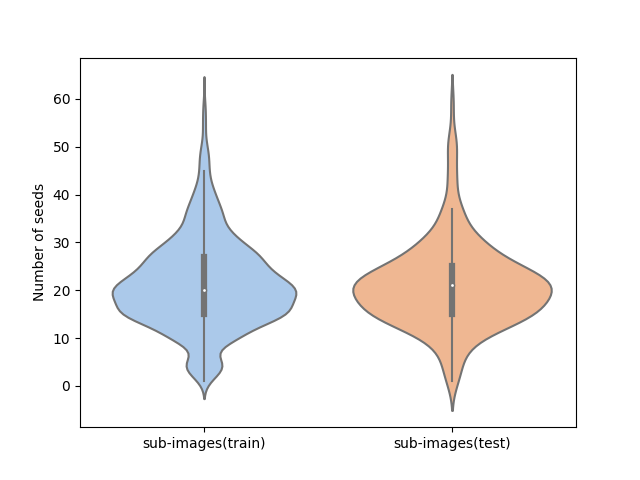* | *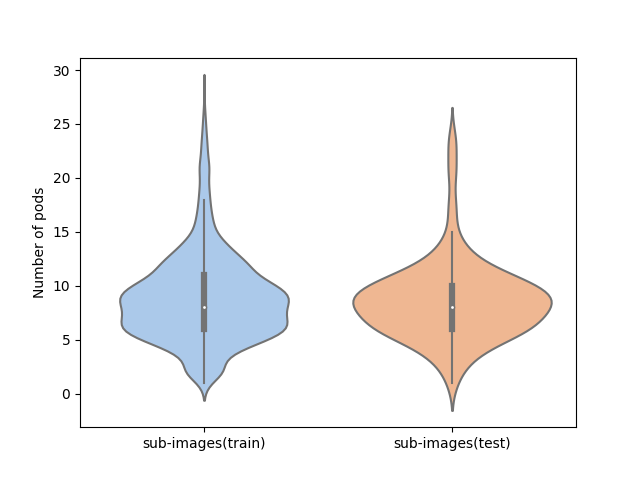* |
| --- | --- |
| (a) | (b) |

*Fig. S3. Distribution of pod and seed counts in the sub-image dataset across training and testing sets. (a) Distribution of seed counts within the training and testing subsets. (b) Distribution of pod counts within the training and testing subsets.*

*
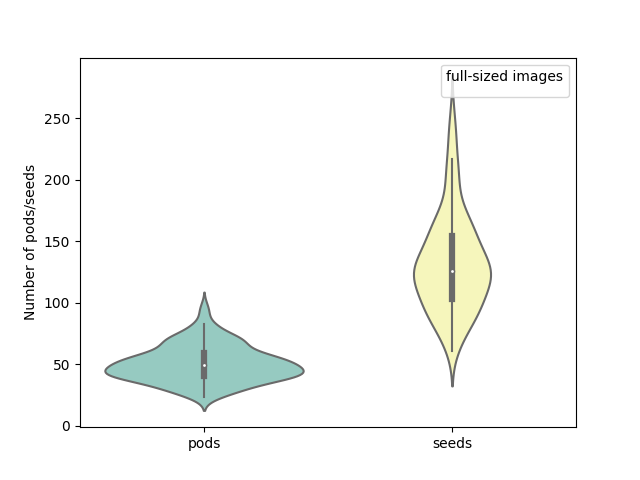
*

*Fig. S4. Distribution of pod and seed counts within the full-sized images testing set.*


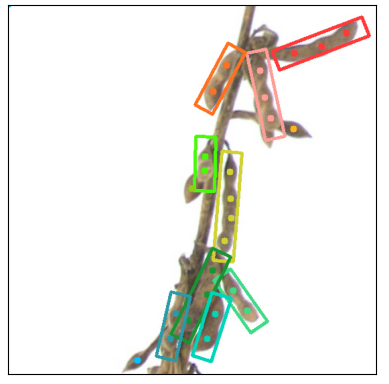


*Fig. S5. For each pod, the position of the bounding box is calculated based on the position of its first seed and its last seed. As can be seen, for a 1-seed pod and a 4-seeds pod, the difference in their bounding box sizes can be very large.*


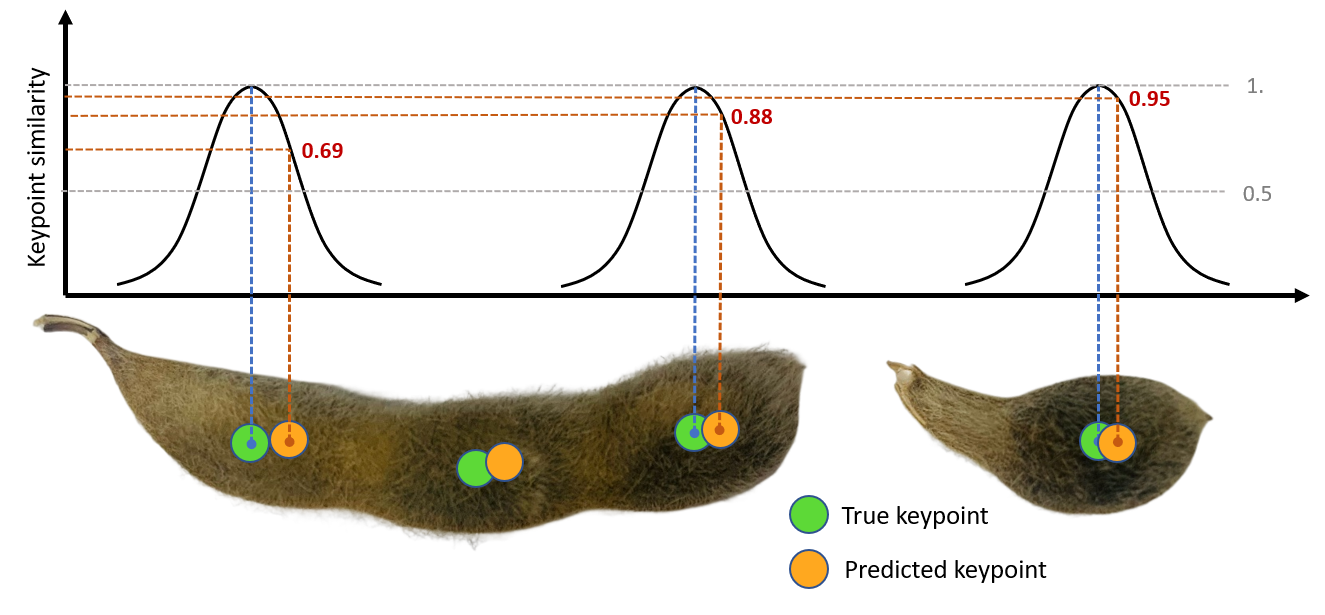


*Fig. S6. Gaussian curves having the same standard deviation for different types of pods.*

*
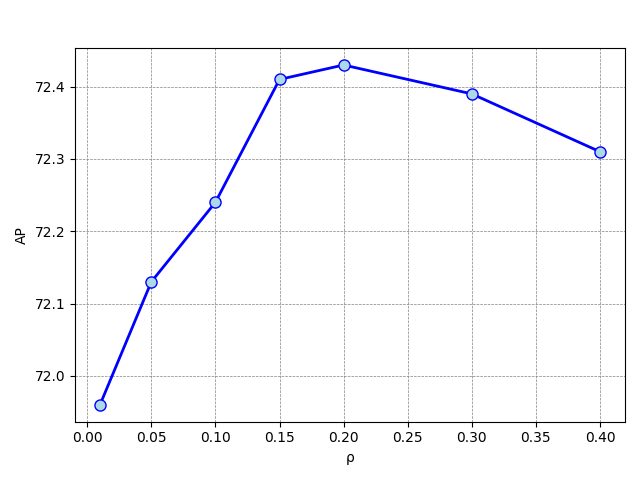
*

*Fig. S7. Ablation studies on loss function and Sprior block.* *The x-axis represents the varying values of the hyperparameter* $\rho$*, while the y-axis depicts the corresponding Average Precision (AP) scores.*
