# Supplementary figures and images for: DEKR-SPrior: An Efficient Bottom-Up Keypoint Detection Model for Accurate Pod Phenotyping in Soybean
Source: Plant Phenomics. 2024 Jun 27;6:0198. doi: 10.34133/plantphenomics.0198 (PMC11209727; doi:10.34133/plantphenomics.0198)

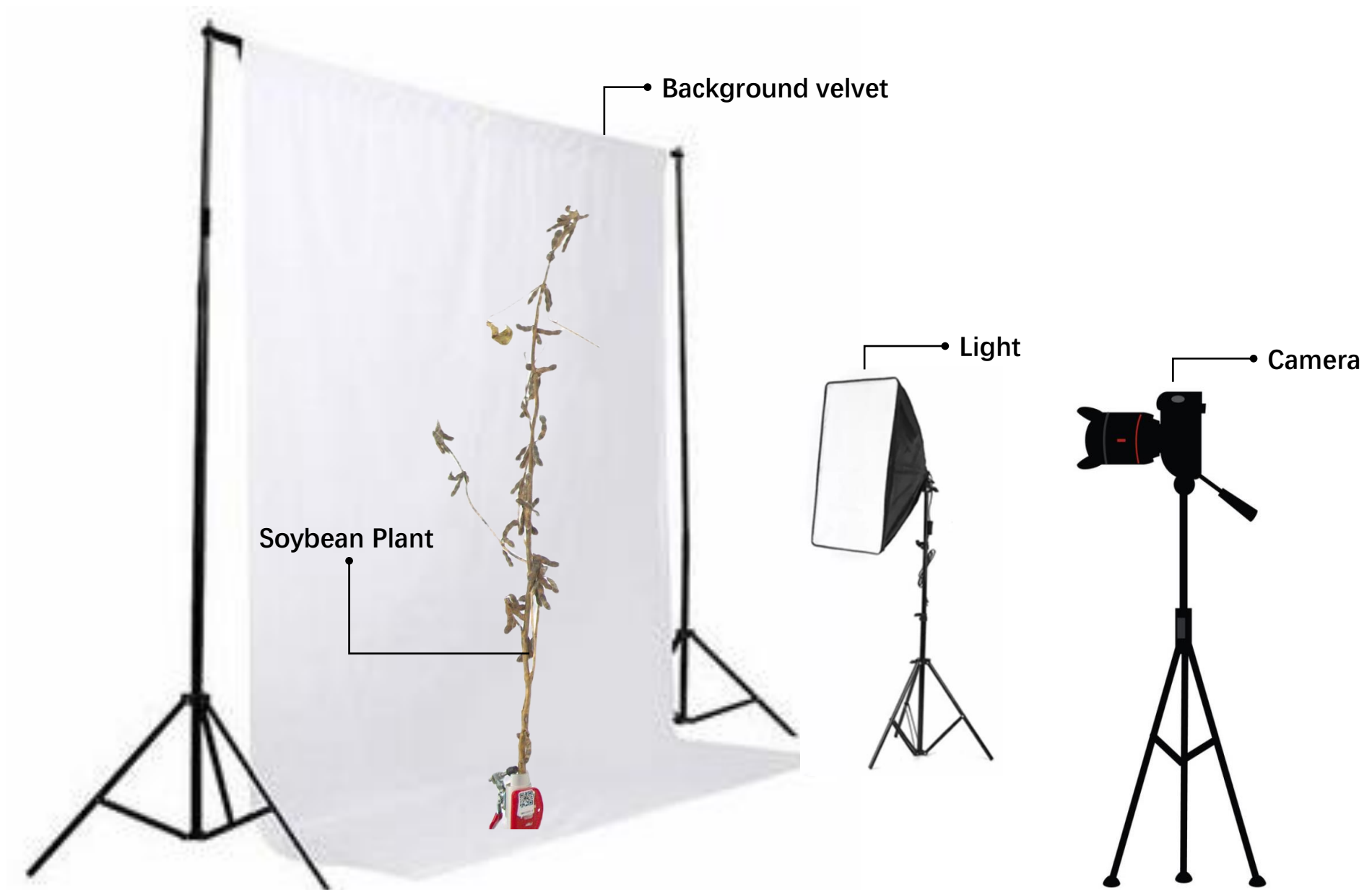

Supplement: Supplementary 1 — Figs. S1 to S7 [file plantphenomics.0198.f1.zip › Fig.S1.pdf]

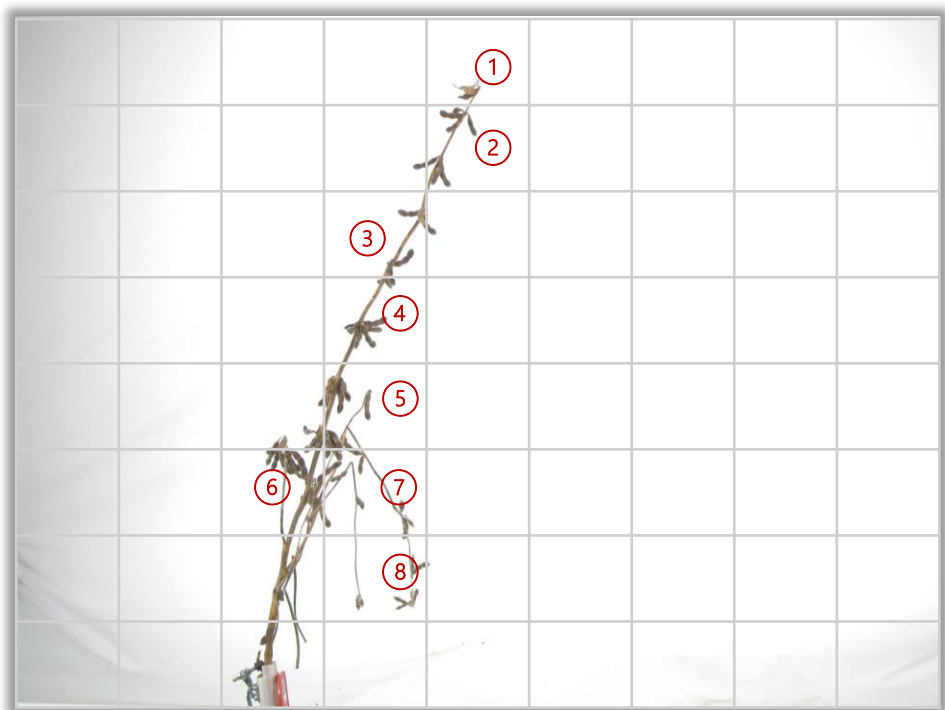

Crop

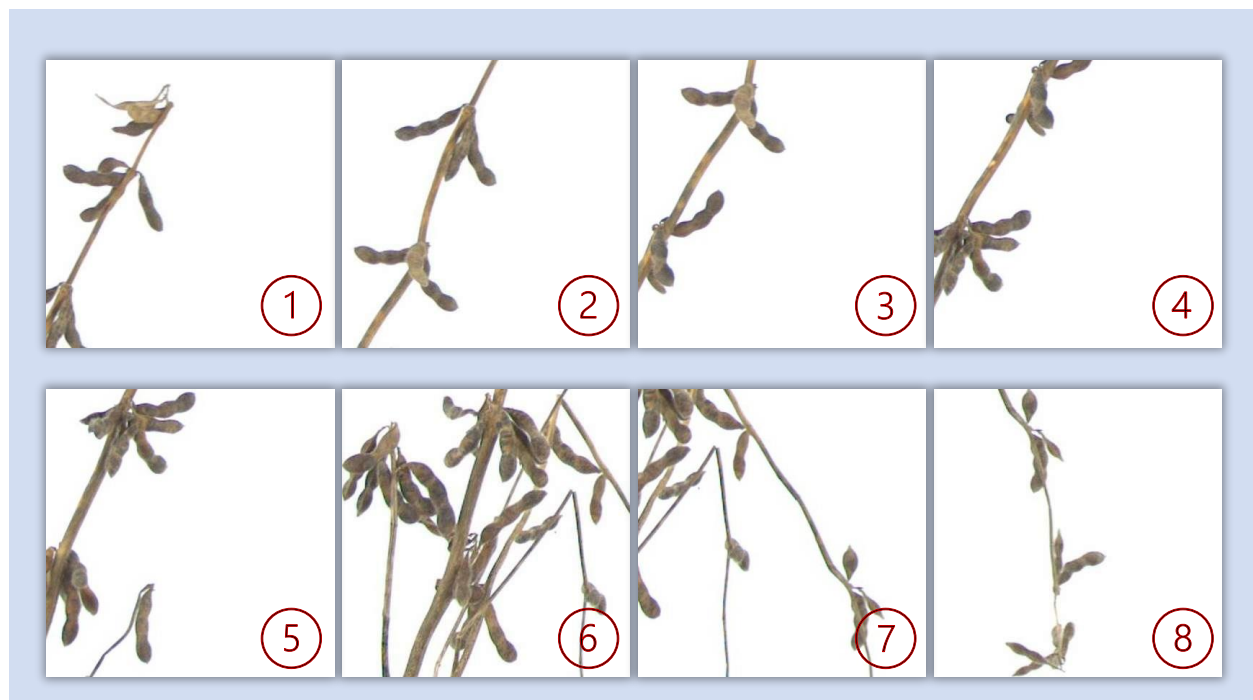

Supplement: Supplementary 1 — Figs. S1 to S7 [file plantphenomics.0198.f1.zip › Fig.S2.pdf]

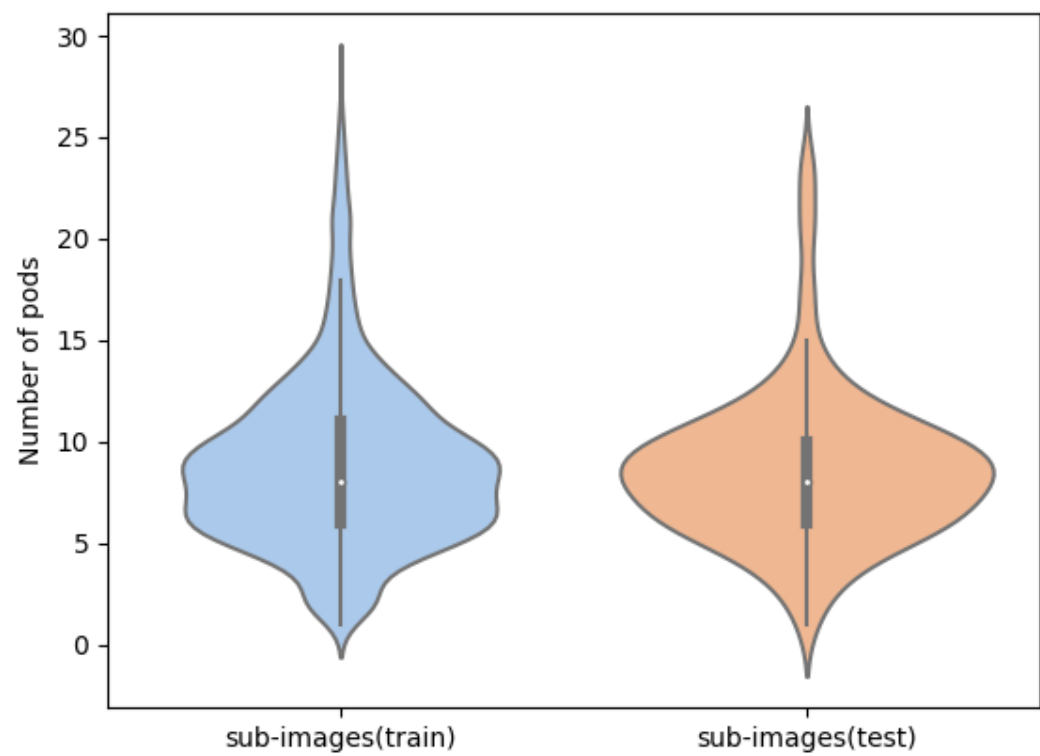

(a)

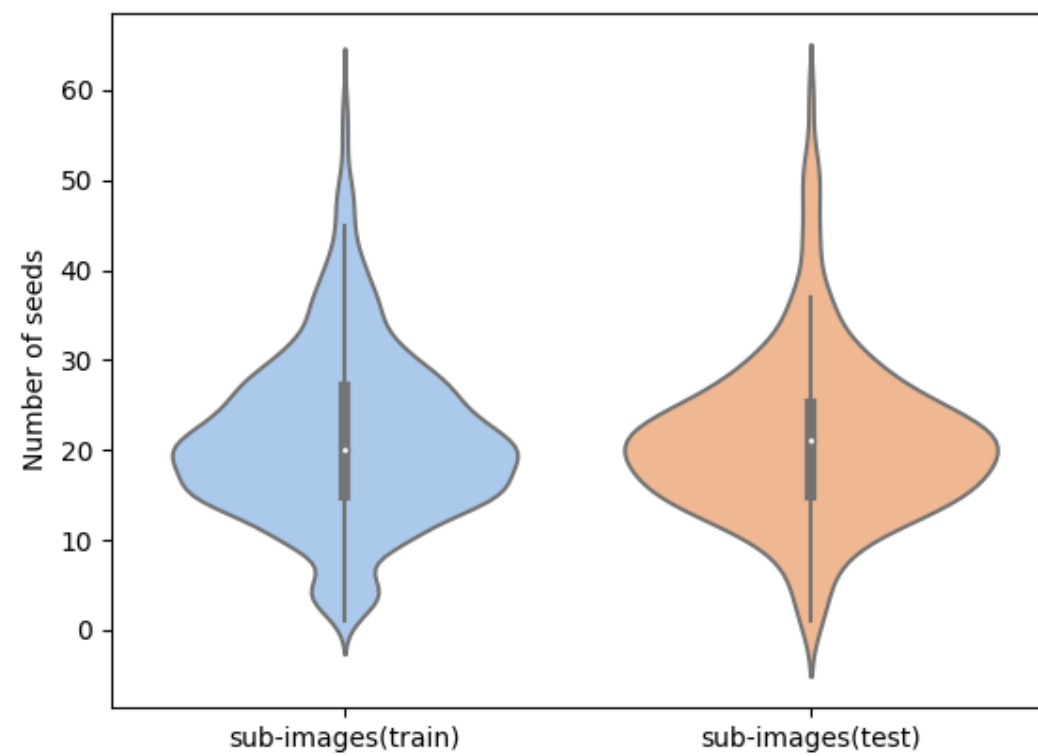

(b)

Supplement: Supplementary 1 — Figs. S1 to S7 [file plantphenomics.0198.f1.zip › Fig.S3.pdf]

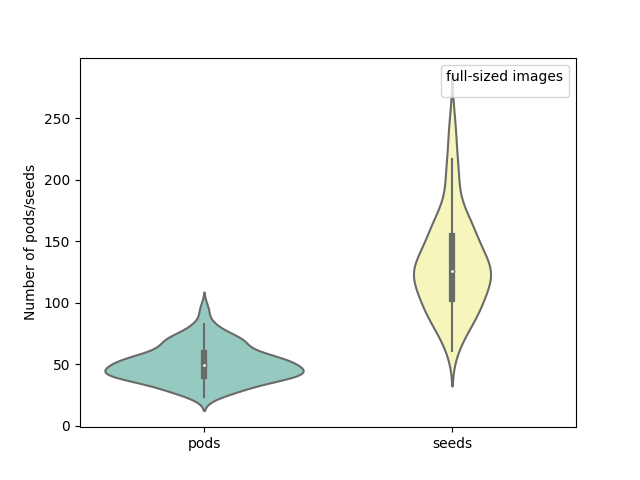

Supplement: Supplementary 1 — Figs. S1 to S7 [file plantphenomics.0198.f1.zip › Fig.S4.png]

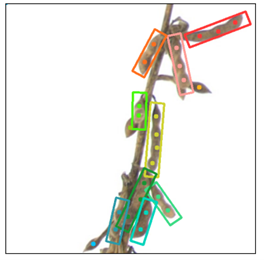

Supplement: Supplementary 1 — Figs. S1 to S7 [file plantphenomics.0198.f1.zip › Fig.S5.png]

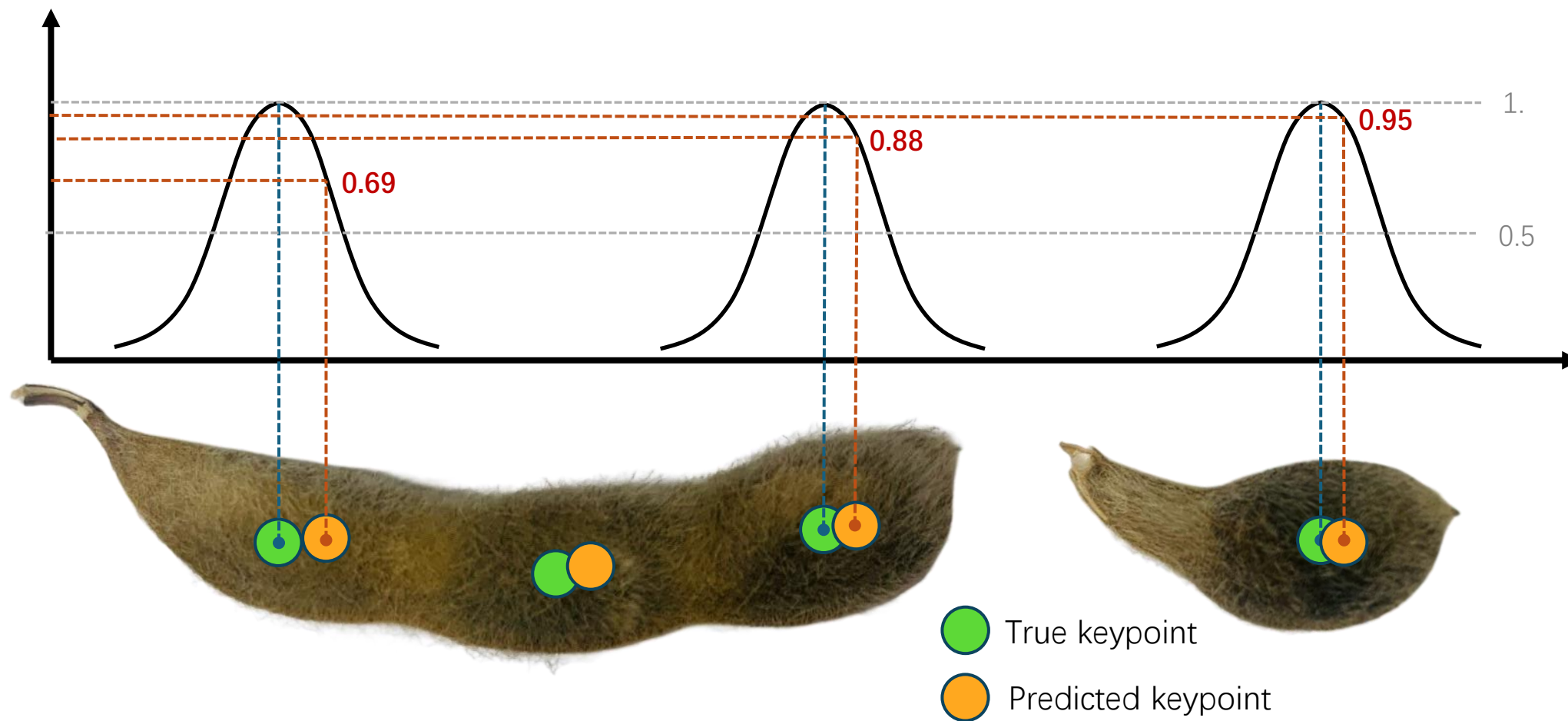

Supplement: Supplementary 1 — Figs. S1 to S7 [file plantphenomics.0198.f1.zip › Fig.S6.pdf]

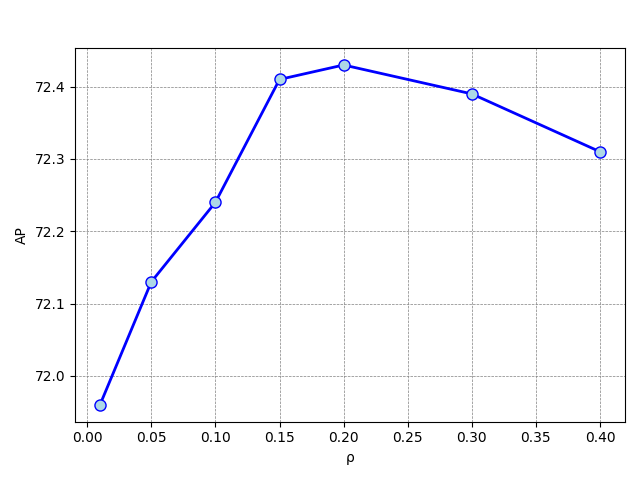

Supplement: Supplementary 1 — Figs. S1 to S7 [file plantphenomics.0198.f1.zip › Fig.S7.png]
